# "Me and Him Is Gradyates of the Same College"

**Published:** 1879-09

**Authors:** W. H. Robinson

**Affiliations:** Suison, Cal.


### "ME AND HIM IS GRADYATES OF THE SAME COLLEGE."

BY W. H. ROBINSON, D. D. S., SUISON, CAL.

"Dad," said a young hopeful to his paternal ancestor, "I can make these old breeches last a while longer, but I am suffering for a bosom pin." Most of us have seen the boy who said this. His head is covered with remnants of last year's hat; feet bare or would be, if washed. His ragged breeches are suspended over one shoulder by a piece of "Mam's" clothes line. He is gazing wistfully at the bosom of of his hickory shirt. A feeling of dejection and wounded ambition steals over him as he views that desolate shirt bosom; and thinks of sparkling gems that glitter in other bosoms; but alas, his is bare. Do not stifle his noble aspirations; give him a few dimes and let him go to Peter Funks, get his bosom pin and be happy. Now, mark you, that boy is honest; that all he lacks is a bosom pin, is a conviction so strong, you can not by any arguments convince him to the contrary, or make him believe that he is not an appropriate subject for a bosom pin. He knows he is. He gets it.

Now as he gazes complacently at his sparkling brilliant that cost him twenty-five cents, he feels that that was the finishing touch that made him a gentleman, and now he is vastly superior to the intelligent schoolboy who has no bosom pin.

As you and I look at the boy, we think how sadly he needs a turkish bath, clean clothes and a few years' schooling. But in his estimation, a boy that can sport a bosom pin is above such minor considerations. He feels away up in the scale of humanity, and is quite sure the bosom pin placed him there and made him the superior of the gentleman or scholar. Our outside view differs materially from his. We see a rude, uncouth ignoramus and a silly gew-gaw.

The boy is a positivist; knows best a bosom pin was all he lacked; that obtained, his education and manhood are complete. That boy grew to be a man. He did not go to school much. He belongs to too high an order of intelligence to be a common laborer or mechanic--lucky for the mechanics—so he chose a learned professsion. Spent a few months in a doctor's or dentist's office; soon knew more than his preceptors; and then discovered that he just lacked one thing to complete his professional erudition, and that was another bosom pin with M. D. or D. D. S. on it. Give him this glittering bosom pin and he possesses every qualification to take rank as a bright particular star in a learned profession.

Professional bosom pins are cheap. Two full courses, three years' reputable practice in lieu of one, and he dons his new bosom pin, and swaggers as he refers to another of his ilk and utters the classic sentence that heads our article: "Me and him is gradyates of the same college." This classical sentence is not mythical. It is the sublime utterance of a veritable titled graduate, displaying his profound scholarship and high professional standing.

Seriously, what does the intelligent public world think of a body of men—if you please, professors in a scientific college. Representatives of a learned profession and the faculty of a college or university, admitting by their diplomas to learned professions men utterly ignorant of the first principles of knowledge? Men who can not write or utter correctly a common sentence in their mother tongue. How proud an alma mater must feel over the offsprings who say, "me and him is gradyates of the same college," or who, as a learned expert, displays his bosom pin by swearing in a court of justice, that "psora sopteunis was not a nerve but a bone.." I am well aware that isolated cases prove nothing, but the vast number of graduates ignorant of the first rudiments of knowledge are not isolated cases. Not one half of the graduates of our dental or medical schools, have received anything like proper literary or mental training. The result is, they can not comprehend the literature of the profession or understand or apply the college curriculum. We might as well expect the laborer's muscle to guide the surgeon's knife or the artist's pencil, as the undisciplined mind to grasp the principles or comprehend the sciences taught. Had the candidates whom our medical and dental colleges matriculate, a common literary and scientific education to start with, they would certainly merit M. D. or D. D. S. did they even get a good elementary training in the branches they are supposed to become proficient in, in two courses of three or four months. But when a lot of boys without an elementary common school education, are for in from six to eight months crammed with big words, and principles that only disciplined minds can grasp, and sciences beyond their mental capacity—what can we expect but alumni whose only claims or evidence of literary or scientific culture, are their professional bosom pins. We have just examined the annual announcement of most of our dental colleges, and the requirements for matriculation are, 1. A good moral character, this is very important, about as important as a pair of trowsers and equally as common. No candidates ever apply to our schools, who have not a good moral character.

Some few of our schools require some common education—one tells what this is, but most are not fastidious on this point—and never ask the matriculant if he has been to school—and can spell "college" or parse "cat." Now these boys, in two courses of three to four months—or an equivilent for one, are taught as follows:

School No. 1. Practical anatomy, visceral, anatmony, regional anatomy, materia medica, therapeutics, chemistry practical histology, histology, physiology, surgery, oral surgery, operative dentistry, mechanical dentistry, pathology and practice, diseases of women, clinics, dissections, etc.

This looks well in print, and is well to the man who has had his mind properly disciplined, to comprehend and investigate such subjects. But what are they to the man without literary training or mental discipline? How can a man extract the cube root who knows nothing of multiplication or division? The supposition is an absurdity. All he knows about them is that "me and him is gradyates of the same college."

Here we have a concern, claiming to be a college, saying "*Omnibus ad quos hac litterae praesentes pervenimit,*" that they have taken an ignorant boy, and in from six to eight months, made him proficient in sixteen scientific branches, that in some cases are above his comprehension, and, to any one of which, disciplined minds could give the whole time in which he is vouched for as having mastered all.

"I guess I had ortoo know sonthing about dentistry," said a new fledged Dr., "I spent a hull year in a dentist's office."

What does the average M. D., or D. D. S., mean? Simply that "me and him" attended two full courses, heard the professors talk, comprehended little of their lectures, and still less of the text books, memorized and plagiarised enough to answer a few common place questions. In the same way produce a school boy composition, call it a thesis—pay the fee, and get the bosom pin.

We are not descrying our medical or dental schools, their curriculi are very good to minds advanced enough to comprehend the sciences taught. Their great mistake, is in matriculating students with no education to start with—and to whom a course in orthography and syntax, would be much more beneficial as a preparatory step toward the practice of medicine or dentistry, than a course in histology and therapeutics. I am well aware that many of the scholars and dentists of the world are M. D's., and that some D. D. S., rank high among them. But these invariably had good mental training and discipline, before they started in professional studies. Many of them do not even wear their bosom pins. You find them stuck away in some little page or "proceedings." When we hear the back-woodsman say "me and him is hunters," the language is no reflection on his abilities as a marksman. But when a titled alumnus of a scientific college has been declared by a learned faculty, to possess scholarship, scientific knowledge, and professional attainments sufficient to give him standing in a learned profession—makes it plain to everybody of common intelligence—that said titled scholar is utterly ignorant of the plainest principles of knowledge, and knows no more about the sciences of his profession, than a Hottentot does about astronomy. What does the intelligent world think of such fools? What of the university, college or faculty, who declares such ignorami learned, and gives them diplomas of scholarship and scientific attainments, that places them by common consent, in some learned profession. Lion skins may cover asses, but do not make lions.

Professional schools should never even matriculate a student, till he has mastered the principles of his mother tongue, and has sufficient knowledge of other languages, to enable him to understand scientific nomenclature. In general, he should possess about the grade of scholarship our average literary colleges require for A. B., or B. S. Professional schools, that make no requirements of this kind, are a disgrace to the intelligence of the age.

We know of nothing that tends so much to degrade our professions—bring their diplomas into contempt, and make their titled alumni a laughing stock, as these, "me and him gradyates," unless it be the diploma mills, that bear the name of college, and give titles to bores—profoundly ignorant of the simplest principles of the sciences, in which their diplomas say they are masters.

The school master is abroad in the land, an intelligent public, looks more carefully at the man's attainments, than at his titles or diploma. Legal enactments never can protect the public from ignorant pretenders, or give professional men true attainments. But schools can do both—their true mission is to do it. Shame on the learned profession, that has to seek legal protection from ignorant outsiders. Professional schools that would expect thorough literary culture, and mental discipline of their matriculants, and then require three or four years studious application to the specialties of any chosen profession; would soon give the world a class of men that would make M. D., and D. D. S., mean more than a piece of parchment, with big words that enables the owner to make a brilliant display of his high scholastic attainments, by saying in the vernacular of his alma mater, "me and him is gradyates of the same college,"

